# The complex relationship between multiple drug resistance and the tumor pH gradient: a review

**DOI:** 10.20517/cdr.2021.134

**Published:** 2022-04-03

**Authors:** Tomas Koltai

**Affiliations:** Former Medical Director of the Hospital del Centro Gallego de Buenos Aires, Buenos Aires 1006, Argentina.

**Keywords:** Multidrug resistance, pH gradient inversion, reversion of the pH gradient, P-gp, pH centered treatment.

## Abstract

Multiple drug resistance (MDR) is the tumor’s way of escaping the cytotoxic effects of various unrelated chemotherapeutic drugs. It can be either innate or acquired. MDR represents the end of the therapeutic pathway, and it practically leaves no treatment alternatives. Reversing MDR is an unfulfilled goal, despite the important recent advances in cancer research. MDR, the main cause of death in cancer patients, is a multi-factorial development, and most of its known causes have been thoroughly discussed in the literature. However, there is one aspect that has not received adequate consideration - intracellular alkalosis - which is part of wider pH deregulation where the pH gradient is inverted, meaning that extracellular pH is decreased and intracellular pH increased. This situation interacts with MDR and with the proteins involved, such as P-gp, breast cancer resistance protein, and multidrug associated resistance protein 1. However, there are also situations in which these proteins play no role at all, and where pH takes the lead. This is the case in ion trapping. Reversing the pH gradient to normal can be an important contribution to managing MDR. The drugs to manipulate pH exist, and most of them are FDA approved and in clinical use for other purposes. Furthermore, they have low or no toxicity and are inexpensive compared with any chemotherapeutic treatment. Repurposing these drugs and combining them in a reasonable fashion is one of the points proposed in this paper, which discusses the relationship between cancer’s peculiar pH and MDR.

## INTRODUCTION

For more than 3000 years, trepanations (for unclear reasons) have been performed, since 2000 BC (and a breast cancer tumor was excised approximately at the same date); up to the 20th century, surgery was the only available treatment for solid tumors. During that time, there was nothing to be done for non-solid tumors. Things started to change in the middle of the last century.

Chemotherapy started in earnest in the 1940s, a decade in which two important advances achieved clinical status. In the first of these events, Louis Goodman and Alfred Gilman created the first alkylating agent as a derivative of the poisonous nitrogen mustard gas, a sibling of the sulfur mustard gas used in WWI^[1]^. The second event had Sydney Farber as the protagonist. He introduced the first anti-metabolite (aminopterin) for acute leukemia treatment in children^[2]^. Interestingly, the title of the first publication by Farber and his associates starts with “Temporary relief…”, thus, incorporating from the first moment one of the main problems of chemotherapy, the limited duration of its benefits. In both cases, the first patients treated by Goodman and Gilman on the one hand and the children with acute leukemia treated by Farber *et al.*^[2]^ on the other hand, the effects of chemotherapy were not long lived and repeat treatments were usually unsuccessful. Chemotherapy came up against its main adversary, resistance. To prolong the beneficial effects and at the same time reduce toxicity, multidrug chemotherapy protocols were introduced - successfully in many cases. Remissions lasted longer and toxicity was reduced. However, resistance and relapse still persisted at the end of the therapeutic path. Patients receiving chemotherapy can in many cases become resistant to previously effective drugs. Unfortunately, resistance is the proof of concept that cancer cells are the most adaptive cells in eukaryotes.

Resistance is the product of two different sources:

(a) The malignant cell itself; and

(b) The stroma and the vascular system.

The most important mechanism is the one in which the cancer cell develops the ability to prevent the drug from entering it or reduces the amount that can enter, simply expels the drug, or can inhibit apoptosis despite the treatment.

**Figure 1:**
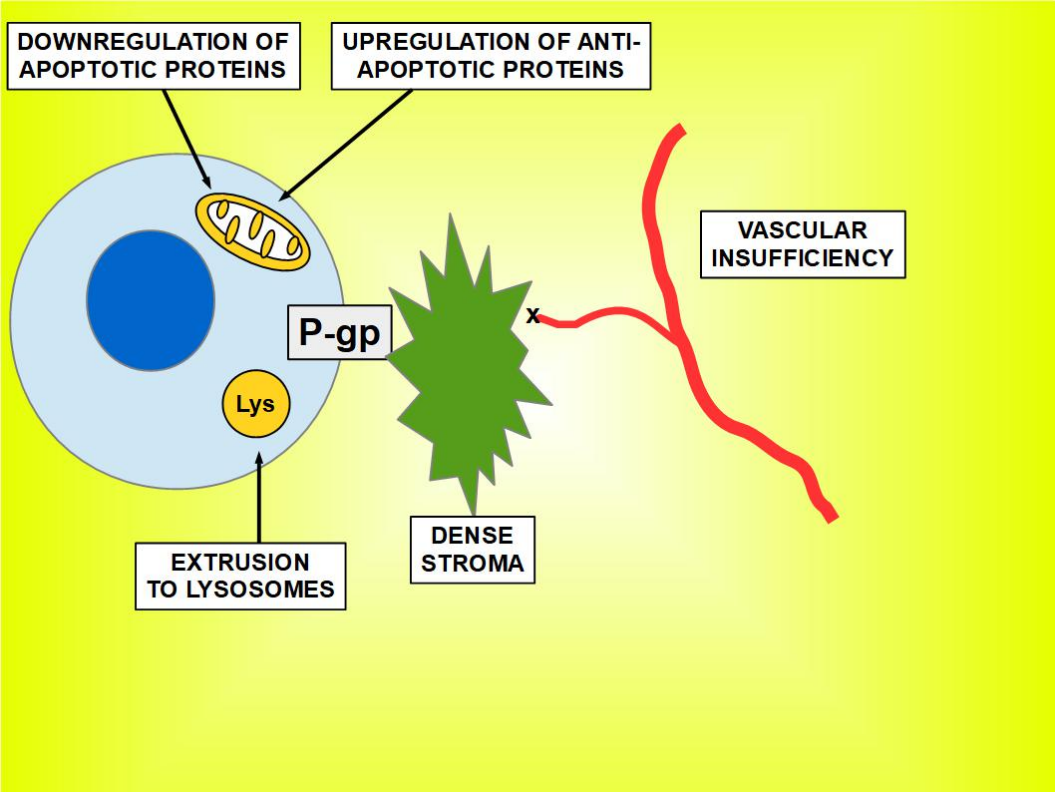
An overview of the main mechanisms of drug resistance. Drug extrusion is represented by P-gp (P-glycoprotein).

The stroma may contribute to resistance through its dense composition (e.g., pancreatic cancer with desmoplastic reaction) or its low vascular supply that decreases drug distribution in the tumor [Figure 1].

A distinction must be made between primary (or intrinsic) and acquired (or secondary) resistance. In the first case, cells are resistant to the drug before they first encounter it. In the latter, acquired resistance, the tumor develops resistance during the course of chemotherapy. This difference implies that, in the second case, tumors evolve from their initial responsive status towards an unresponsive one. The main evolution consists in upregulating the expression of some specific proteins of the ATP binding cassette family (ABC), namely P-glycoprotein (P-gp, also known as MDR1 or ABCB1), multidrug associated resistance protein 1 (MRP1 or ABCC1), and breast cancer resistance protein (BCRP or ABCG2).

The introduction of novel targeted therapies in the last twenty years was expected to dramatically change the resistance problem. That did not happen. Targeted treatments improved survival and progression-free periods in many different cancers - in some, a cure was even achieved - but resistance to treatment remained little changed in most cases.

In 1958, Burchenal and Holmberg were the first to study the short-lived remission achieved with anti-metabolites in the treatment of leukemia at a cell level^[3]^. They expressed the prevailing concept at that moment - a random mutation induced by the drug - but they also established that there were many other biochemical mechanisms leading to resistance.

There is a special form of resistance consisting of invulnerability to many unrelated drugs that were never administered to the patient. This is known as MDR, a situation that severely limits the therapeutic options and consists of increased efflux of drugs**. **This mechanism was first proposed by Keld Danø, in 1973, as probably the main mechanism ^[4]^. This drug extrusion hypothesis was followed some years later by the discovery of P-glycoprotein ^[5]^ and other MDR proteins such as multidrug- associated resistance protein 1 (MRP1) ^[6]^ and BCRP^[7]^, proteins that were not known when Danø proposed his theory. This meant that the extrusion culprits were finally identified.

### Multiple drug resistance

Resistance to a drug must be differentiated from multidrug resistance. In the first case, a mutation or clonal evolution can be the cause, supposing that the malignant cells are responsive to other chemotherapeutic drugs. It is the lack of response to many different drugs that signals towards MDR, especially if these drugs were never administered before. In this last case, the most probable cause is the increased expression of one or more of the MDR proteins. Therefore, it is cancer’s resistance to many different and unrelated chemotherapeutic drugs that defines MDR, which may be intrinsic (primary) or acquired after chemotherapy. The lack of specificity is still one of the issues that is hard to explain. The causes of MDR are multiple, from poorly drug vulnerable stem cells to clonal evolution, stromal barriers, and mutations. It is not the aim of this paper to discuss MDR mechanisms but to focus exclusively on those related to pH alterations. When chemotherapy initially works, it is able to induce apoptosis in many of the malignant cells. However, tumors are very heterogeneous ^[8,9]^ and not all malignant cells will be eliminated with the treatment. Those that survive repeated cycles of cytotoxic medication are either intrinsically resistant, or they are located in inaccessible parts of the tumor, such as very hypoxic niches without blood supply or in the middle of dense connective tissue with high interstitial pressure impeding circulation [Figure 1]. The non-resistant cells are gradually killed, while the surviving resistant cells will thrive and progress and, ultimately, fully replace the “weaker” cells, thus heralding the relapse. This is a typically Darwinian evolution where chemotherapy is the selective force for the survival of the fittest.

For extensive reviews on MDR, read the works of Gillet* et al.*^[10]^, Rascio *et al.*^[11]^, Mansoori *et al.*^[12]^ Jayaraj *et al.*^[13]^, Ruan *et al.*^[14]^, Aleksakhina *et al.*^[15]^, and Vasan *et al.*^[16]^.

This paper does not analyze MDR causes in depth, which is beyond the scope of the review, but rather focuses on the relationship between the tumor dysregulated pH and MDR and explores new therapeutic avenues in this regard.

### pH deregulation in cancer

It has been known since the work of Otto Warburg ^[17]^ in the 1920s that tumors are acidic due to excessive production of lactic acid as a consequence of high glycolytic flux and downregulation of mitochondrial oxidative activity. Between the 1920s and the beginning of the 1970s, it was believed that there was no difference between intra- and extracellular pH. Thus, if the tumor was acidic, this concept included both sides of the cell membrane. Only in the late 1970s did it become evident that acidity was limited to the extracellular matrix, while in the intracellular milieu, pH was either unchanged or increased, compared with normal cells. The long time it took to discover the pH differences on the two sides of the cell membrane was due to the lack of adequate instruments that could accurately gauge intracellular pH [Table 1]. The awareness of different intra- and extracellular pH levels also led to the discovery of the channels, exchangers, transporters, and enzymes located on the membrane, which are in charge of maintaining this pH differential (gradient).

**Table 1:** pH in different compartments in normal and cancer cells

**Table 1**	**Normal cell**	**Cancer cell**
Extracellular (EC) pH (pHe)	7.30-7.35	6.4-7.0
Intracellular (IC) pH (pHi)	7.2	7.25-7.50
pH gradient	EC→IC	IC→EC

In Table 1, the direction of the arrow indicates the pH gradient, and we can easily see that in cancer, the gradient follows exactly the opposite direction compared with non-malignant counterparts. This is the inversion of the pH gradient, a process found in all tumors, which is fundamental for cancer cell survival and progression. This means that in cancer, extracellular pH becomes acidic and intracellular pH becomes more alkaline.

Extracellular acidity, known for a long time, and the more recently discovered intracellular alkalinity were merely considered as a consequence of cancer metabolism and, to a certain extent, innocent bystanders. This notion started to change in the mid-1980s, when researchers found more and more evidence showing that the inverted pH gradient represented an important advantage for growth, proliferation, migration, and invasion. Furthermore, in 2000, Reshkin *et al.*^[18]^ showed that the first step in cellular transformation consisted in an increase of intracellular pH as a consequence of the enhanced activity of a specialized membrane channel, sodium bicarbonate exchanger 1 (NHE1). This exchanger, one of the main players in intracellular pH homeostasis, has the ability to export hydrogen ions (H^+^, protons) while importing Na^+^ from the matrix into the cells.

Reshkin *et al.*^[18]^ went one step further; they inhibited NHE1 with a drug that has been in clinical use for more than fifty years, amiloride. Interestingly, NHE1 inhibition impeded malignization. This was clear proof of the importance of pH in cancerization.

Intracellular and extracellular pH are discussed separately below, even though they are part of the same process of pH deregulation. The membrane between both compartments is the tool that keeps this different pH alive. The membrane is not a simple and passive boundary, but rather it is an active player that maintains a different environment inside and outside the cell. This is in part achieved by channels, exchangers, transporters, and enzymes spanning through it.

### Extracellular and intracellular pH (pHe and pHi)

**Figure 2:**
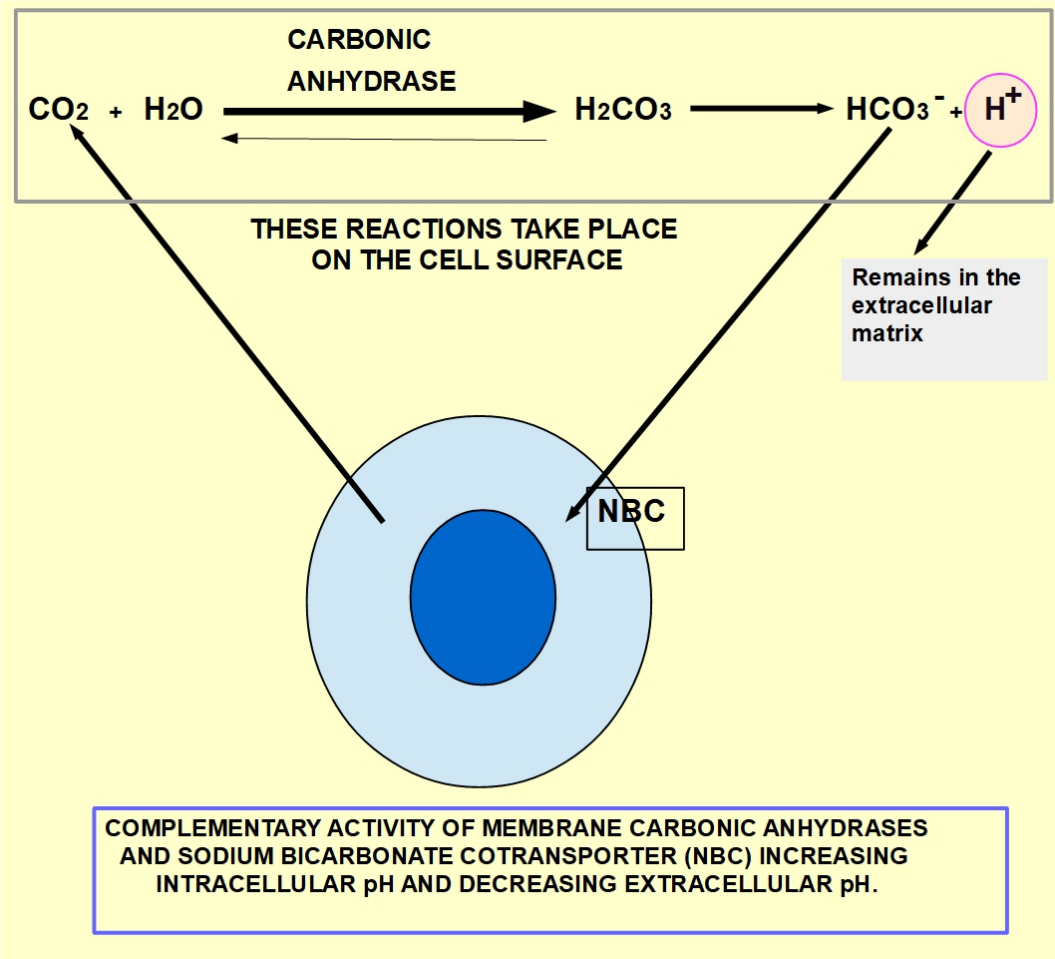
CO_2_ of metabolic origin diffuses to the cell surface where it is hydrated to carbonic acid, which spontaneously ionizes to form a proton and a bicarbonate molecule. While the bicarbonate is reintroduced into the cell by NBC, the proton remains in the extracellular matrix, contributing to its acidification. NBC: Sodium-bicarbonate cotransporter.

Normal extracellular pH, which is very close to the blood pH, decreases by around 8–10% in cancer tissues. It goes from 7.35 (normal tissues) to roughly 6.8 (malignant tissues). In some cases, it becomes even more acid. This is the result of different events:

(a) Increased CO_2_ production that produces carbonic acid on the cell surface and immediately ionizes, generating a bicarbonate ion that is reintroduced into the cell and a proton that remains in the extracellular matrix [Figure 2]. Two proteins located in the cell membrane participate in this process, namely membrane carbonic anhydrases (isoforms CAIX and CAXII) and sodium-bicarbonate cotransporter (NBC). CAIX is usually overexpressed in many hypoxic tumors^[19-22]^. CAIX is so closely associated with hypoxia that many authors consider its overexpression as a hypoxia marker^[23-29]^.

CO_2_ abandons the cell simply by diffusion. When it reaches the cell surface, there is a tandem activity on CO_2_ carried out first by membrane carbonic anhydrase IX or XII and then by NBC [Figure 2].

Since Warburg’s work and until 1999, lactic acid was considered the main culprit of extracellular acidosis in cancer. Seminal research by Newell *et al.*^[30]^ showed that eliminating lactic acid production in malignant cells only minimally modified extracellular acidosis; thus, lactate is not the main and sole origin of a low pHe. It seems that CO_2_ production is equally important in pHe descent. Malignant cells produce large amounts of CO_2_ through the very active pentose phosphate pathway and fatty acid beta-oxidation.

**Figure 3:**
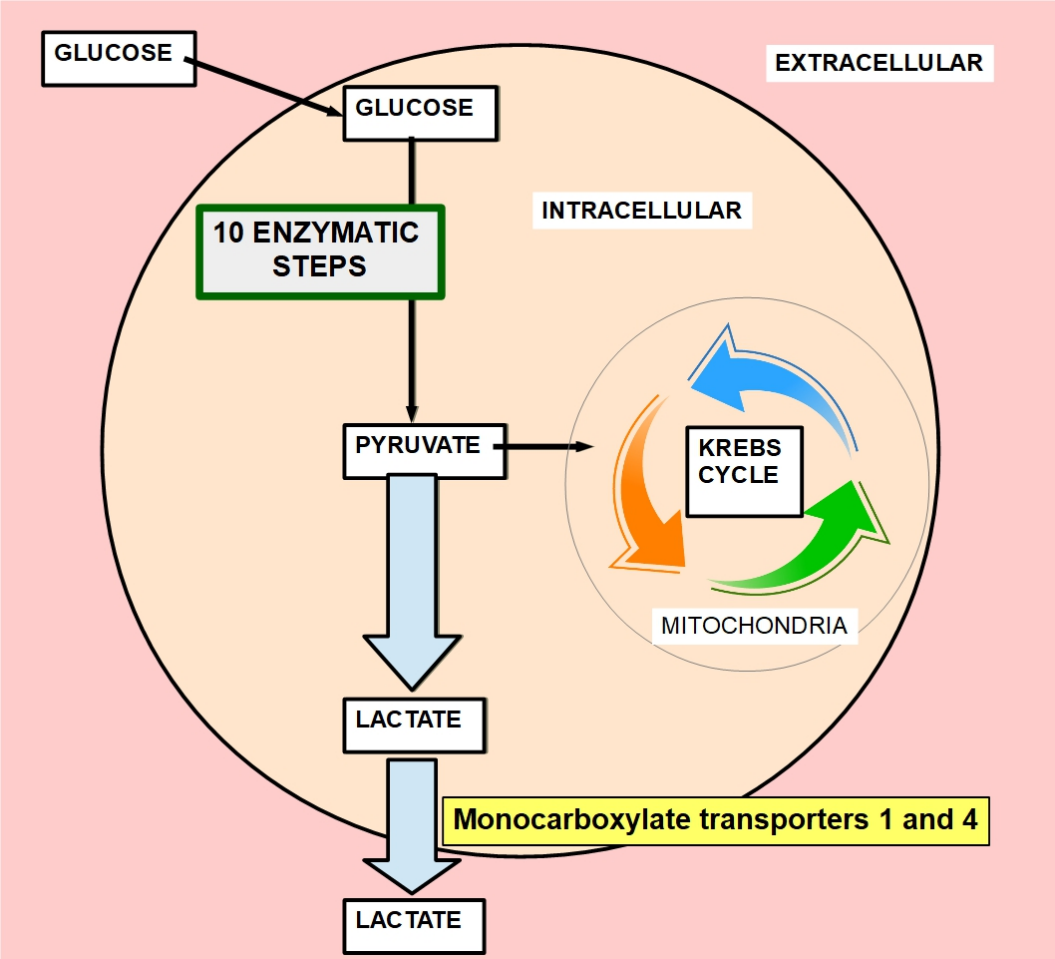
Origin of lactate production. Normal cells do not use the glycolytic pathway beyond pyruvate that goes into the Krebs cycle associated with coenzyme A and converted into acetyl-CoA. Malignant cells follow the glycolytic pathway ending in lactate that is extruded by the activity of monocarboxylate transporters. Lactate cannot stay inside the cell because it would decrease intracellular pH to life-threatening levels, thus it must be swiftly exported. This soft-spot of cancer metabolism transforms MCTs into valid targets^[31-34]^.

(b) Increased lactic acid production is extruded from the cell by specialized membrane transporters such as monocarboxylate transporter 1 (MCT1) and monocarboxylate transporter 4 (MCT4). Figure 3 shows the origin of lactic acid from the glucose metabolism, which is strongly deviated towards the glycolytic pathway instead of the oxidative pathway of the Krebs cycle. MCTs can carry monocarboxylates in general; they are not exclusively dedicated to lactate transport. In cancer, these transporters are overexpressed on the cell membrane^[35-38]^. Hao *et al.*^[39]^ found that there was an association of CD44, CD147, MDR1, and MCTs expressions with prostate cancer progression and drug resistance.

(c)There is the extrusion of intracellular protons through NHE1, vacuolar ATPase proton pumps.

(d)There is the extrusion of protons through endosomes that become exosomes or simply release their acidic cargo into the extracellular matrix. Endosomes can follow two pathways: (a) transformation into lysosomes; and (b) becoming carriers of compounds that will be extruded from the cell, such as protons, proteolytic enzymes, etc.

(e)Debris of stromal and tumoral cell death are produced, whether by hypoxia, invasion, chemotherapy, or radiotherapy. Necrotic cells are able to release acidic intracellular compounds, while apoptotic cells are engulfed by macrophages and there is no intracellular content released into the matrix.

Reducing extracellular pH simultaneously increases intracellular pH because the mechanism is essentially based on protons released from inside the cell and into the matrix.

Matrix acidification has advantages for malignant cells because acidity:

• Decreases and inhibits immunological attacks on the tumor;

• Activates proteolytic enzymes needed for invasion; and

• Stimulates migration at the invadopodium level,

As mentioned above, intracellular pH increases through the loss of protons towards the matrix and the import of bicarbonate. This situation is also advantageous for the malignant cell because it:

• Increases the activity of glycolytic enzymes, thus enhancing glycolytic flux that allows a higher biosynthetic activity;

• Increases proliferation; and

• Decreases the possibility of apoptosis.

## RELATIONSHIP BETWEEN DEREGULATED pH AND MDR

The relationship between pH and MDR has major participation in the resistance process, as is discussed below. We do not have the evidence to maintain that MDR would not be possible without deregulated pH; however, there are many findings that hint towards this hypothesis. At this point, we are convinced that restoring the normal pH gradient should be an integral part of MDR targeting.

The relationship between extracellular pH and MDR has been extensively studied and is represented by a phenomenon called ion trapping.

### Ion trapping

Weakly basic chemotherapeutic drugs, such as doxorubicin, cannot enter the hydrophobic cell membrane because they undergo ionization in the acidic tumor extracellular environment. The lipid bilayer cell membrane is semipermeable, meaning that, while it allows fat-soluble non-ionized moieties to enter, it is poorly permeable to ionized water-soluble molecules. Anthracyclines and vinca alkaloids are weak basic drugs that are ionized by the microenvironmental acidity, thus substantially reducing their access to the cell^[40-42]^.

There are many reports that serve as proof of concept regarding the relation between ion trapping and extracellular acidity:

• Reducing extracellular acidity with sodium bicarbonate, mitoxantrome cellular penetration was increase^[43]^.

• Gu *et al*.^[44]^ developed a fluorescent probe based on dihydroberberine that showed that ionized berberine had a substantially lower cell penetration than the non-ionized form.

• Proton pump inhibitors, which increase extracellular pH, decrease drug resistance^[45]^.

### Endosomal ion trapping

Martinez-Zaguilán *et al.*^[46]^ described the trapping of chemotherapeutic drugs in cellular endosomes. A new membrane is rapidly formed around the drug molecules, and these endosomes have a high V/ATPase proton activity, meaning that their interior is highly acidic. According to our criteria, drug release from the endosomes is inhibited by a mechanism similar to ion trapping. That is, the acidic interior ionizes weak basic drugs, impeding their transit through the membrane.

### Extracellular acidity induces P-gp expression

This mechanism is independent of ion trapping and consists of the increased P-gp expression when extracellular pH becomes acidic^[47-50]^. The mechanism that leads from extracellular acidity to increased P-gp proteins has not been fully clarified as yet.

### Intracellular alkalinity and its effects on MDR

The intracellular milieu and its sub-compartments, such as mitochondria, Golgi, endoplasmic reticulum, and endosomes, including autophagosomes and lysosomes, all have different pH, which is set according to the needs of the metabolic processes taking place in them:

• Cytoplasm is the site of glycolysis and fatty acid synthesis.

• Mitochondria are the site of the Krebs cycle, the electron transport chain, and lipid beta-oxidation.

• Golgi and endoplasmic reticulum mature proteins and secretions.

• Autophagosomes recycle organelles and other nutritional agents.

• Lysosomes and endosomes are very acid, degrade biological products, and intervene in the maturation of proteolytic enzymes.

Each of these activities requires a different optimum pH, and the cell’s homeostatic machinery maintains these different pHs (gradients) through membranes, e.g., cytoplasmic pH around 7.2, mitochondrial pH around 8, and lysosomal pH below 5.5.

Cell proliferation requires an alkaline cytoplasm, slightly above that of the resting cell.

In an extensive phylogenetic review, Busa and Nucitelli^[51]^ showed that almost all species increase their intracellular pH before replication. There were also some minor exceptions. This led the authors to consider intracellular pH as a signaling mechanism. High intracellular pH does not seem to be an indispensable mechanism but rather a facilitator for cell replication.

Drug-resistant cells were found to over-express or increase the activity of some of the membrane proteins discussed above^[52]^:

• A subunit of a vacuolar H+-ATPase proton pump^[53]^; and

• NHE1 (sodium hydrogen exchanger 1)^[54-56]^.

**Figure 4:**
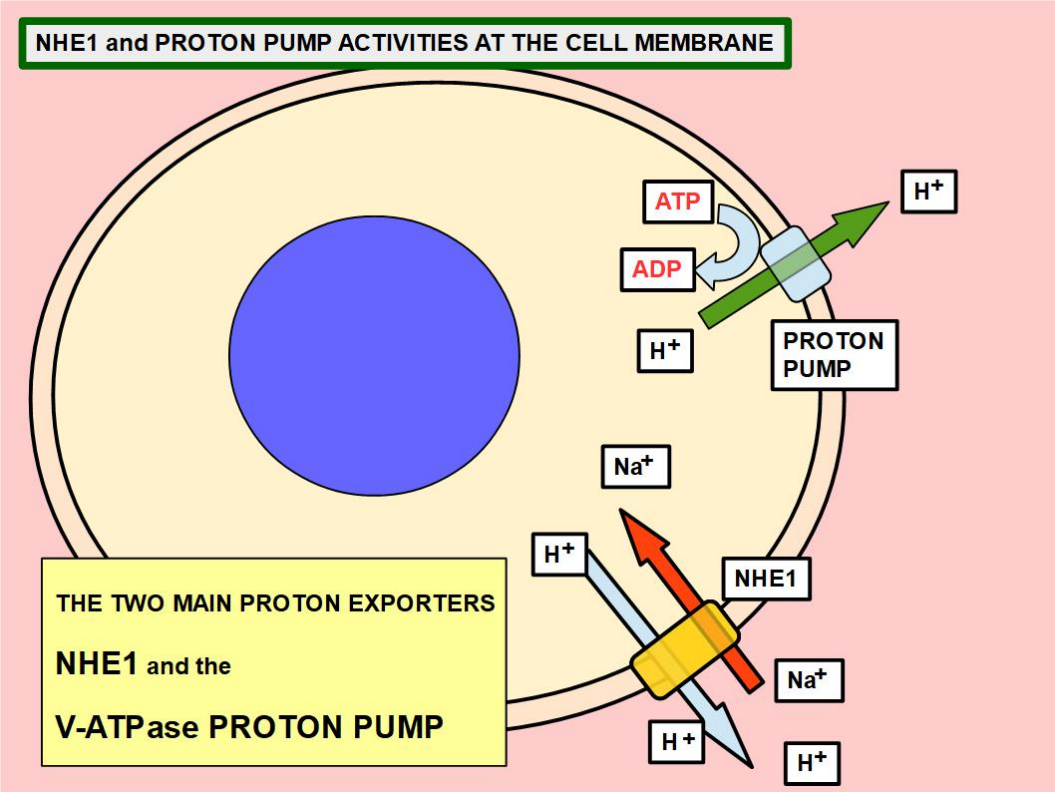
Increase of protons in the extracellular matrix originates mainly from the extrusion of intracellular protons by NHE1 and the proton pump.

Proton pumps and NHE1 [see Figure 4] are directly related to the pH gradient inversion: increasing intracellular alkalinization and decreasing extracellular pH. P-gp has a direct connection with intracellular and extracellular pH because:

• Intracellular acidification downregulates P-gp^[57]^; and

• Extracellular acidity increases P-gp expression by up to 5-10-fold *in vitro *^[58]^.

This particular P-gp/pH relationship may explain some cases of intrinsic resistance, where there was no previous contact with any chemotherapeutic drug.

### Apoptosis requires intracellular acidosis

The main objective of chemotherapy consists in inducing the programmed death (apoptosis) of malignant cells^[59,60]^. If this objective is not achieved, the tumor is resistant^[61]^. Tumors have the ability to suppress apoptosis to a certain extent^[62]^. There are many different modes for achieving resistance to apoptosis. One of them is increased signaling through the pro-survival PI3K/AKT pathway. This is usually associated with, or may produce a high expression of, the anti-apoptotic Bcl2 proteins.

For example, high expression of the anti-apoptotic Bcl2 protein induces an MDR phenotype in lymphoma cells^[63]^. Apoptosis is favored by low intracellular pH and impeded when it is high^[64]^. There is abundant evidence showing that the intracellular decrease of pH is favorable for apoptosis:

• Deoxyribonuclease II is an essential enzyme in programmed cell death and requires an acidic environment for its action^[65]^.

• The first step in apoptosis consists of cytoplasmic acidification mainly due to proton export from mitochondria, thus alkalinizing this organelle and releasing cytochrome C^[66]^.

• Maximal activation of caspases is only achieved with acidic cytoplasm.

• Pharmaceuticals that decrease intracellular pH induce apoptosis. This is the case of lansoprazole (inhibits the proton pump)^[67]^, salinomycin^[68]^, and lonidamine. The antibiotic salinomycin has shown the ability to decrease the efflux of doxorubicin as well as other chemotherapeutic drugs in MDR^[69-72]^ through P-gp inhibition^[73]^. In addition to these effects, it also has anti-tumor activity independently of MDR inhibition^[74-82]^. However, we presume that many of the salinomycin’s anticancer effects are directly related to intracellular acidification. Conversely, P-gp has antiapoptotic effects^[83]^, and prolonged intracellular acidification decreases P-gp expression^[84]^.

## PROOF OF CONCEPT OF THE pH–MDR RELATIONSHIP

It has been known since 1990 that multidrug-resistant cells have a higher pH than their non-resistant counterparts^[85]^. This pH is higher on average by 0.1-0.2 and has been found in many different malignant cell lines. Although this is not a universal finding, it is quite frequent. There is also evidence that the increased intracellular pH is partly due to increased NHE1 activity.

MDR1 expressing cells were resistant to complement-mediated cytotoxicity; however, interestingly, malignant cells not expressing MDR1 that were manipulated to increase their pHi also showed similar resistance to complement-mediated cytotoxicity. Complement deposition in the cell membrane was reduced and delayed in both types of cells to a similar magnitude^[86]^.

Belhoussine *et al.*^[87]^ confirmed the higher intracellular pH in MDR cells. They also found that these cells had more acidified vesicles, which were more acidic than their sensitive counterparts. According to the authors, these findings suggest that non-MDR cells have a lesser ability to remove protons.

Interestingly, some publications showed the ability of amiloride, an NHE1 inhibitor and cell acidifier, to reverse both the pHi increase and MDR^[88,89]^.

Hamilton *et al.*^[90]^ showed that verapamil, a classic P-gp inhibitor, lowered pHi, but they considered these two factors unrelated.

In 1997, Robinson *et al.*^[91]^ found that the expression of MDR1 had the ability to delay apoptosis in fibroblasts transfected with the MDR1 gene and exposed to cytotoxic substances, colchicine in particular. The sole increase of pHi in non-transfected cells was able to induce a similar apoptotic delay. This finding suggests at least two things:

(1) Apoptosis requires low pHi; and

(2) One of the mechanisms used by MDR to oppose apoptosis is to maintain the increased pHi.

What remains to be seen is whether high pHi is a facilitating mechanism for resistance to apoptosis or a causal one. This report is in line with that by Weisburg^[86]^ and supports the idea that increased pHi by itself can induce an MDR phenotype.

MDR has a “protective” effect against caspase-dependent apoptosis. Inhibition of P-gp with specific antibodies increased drug- or Fas-mediated apoptosis through caspase activation in drug-resistant cells^[92]^, and activating caspases requires an acidic cytoplasm. Thus, protection from apoptosis seems to be a synergic activity of MDR proteins plus intracellular alkalinity.

The pH gradient inversion, with its intracellular alkalinity and without MDR proteins, has shown that it can prevent intracellular accumulation of chemotherapeutic agents. In this regard, Simon *et al.*^[52]^ showed that intracellular alkalinity can substantially decrease accumulation and modify the intracellular distribution of weak alkaline chemotherapeutic drugs without any efflux mechanism in place. Intracellular alkalinity can prevent drugs from binding to their targets. Therefore, it is not only the extracellular ion-trapping effect that impedes the activity of weak alkaline drugs. In the experiments performed by Simon *et al.*^[52]^, non-resistant myeloma cells had a significantly lower intracellular pH compared with myeloma chemo-resistant cells (7.1 *vs.* 7.45).

Proton pump inhibitors have shown the ability to decrease or inhibit the MDR phenotype^[93]^. Cisplatin treatment increased V-ATPase proton pump expression, and pHi was significantly increased in cisplatin-resistant cells. The DNA-binding ability of cisplatin was significantly enhanced in a more acidic pHi, suggesting that cisplatin’s cytotoxicity was modulated by pHi. Proton pump inhibitors such as bafilomycin could synergistically increase cisplatin’s cytotoxicity. These findings show that pHi is a key player in cisplatin’s effects^[94]^. Unfortunately, bafilomycin cannot be used in clinical practice due to its toxicity.

Thiebaut *et al.*^[95]^ confirmed the higher pHi in resistant cells compared with their non-resistant counterparts. They performed an experiment in which they raised the extracellular pH and found that non-resistant cells maintained a pHi around 7, but the resistant cells markedly increased their pHi. pHi did not increase when they treated resistant cells with P-gp inhibitors, and they suggested that P-gp also behaves as a proton exporter.

Hoffman *et al.*^[96]^ showed that the level of resistance in MDR cells was correlated with two other parameters, namely pHi and low membrane electrical potential. Resistance was induced when they increased pHi in non-resistant cells. Furthermore, membrane depolarization also conferred a mild chemoresistance without any P-gp participation.

NHE1 is the main proton exporter, albeit not the only one, and it is overexpressed/over-active in cancer cells^[97]^. This is particularly so in resistant cells^[97]^. Cariporide is a powerful experimental NHE1 inhibitor. Interestingly, cariporide, which acidifies cytoplasm, is also able to sensitize resistant-breast cancer cells to doxorubicin^[54]^.

NHE1 inhibition with cariporide reversed imatinib resistance in BCR-ABL-expressing leukemia cells^[98-100]^, and NHE1 knockdown sensitized malignant cells to cisplatin-induced apoptosis^[101]^. Inhibiting proton extruders, among them NHE1, increased doxorubicin’s cytotoxic effects on breast cancer cell lines^[102]^.

pH regulation implies the participation of many players, in addition to NHE1. Membrane carbonic anhydrases are among these participants. Interestingly, by inhibiting membrane CAs, MDR can be reversed^[103-108]^.

The inverted pH gradient has roles at both ends of the gradient: while cytoplasmic acidification downregulated P-gp^[109]^, extracellular acidity upregulated it^[110]^. This finding explains why it is not enough to act pharmacologically on one of the components of pH deregulation; both need to be addressed.

Despite all the evidence supporting the idea that high pHi is an indispensable condition for the MDR phenotype, Young *et al.*^[111]^ showed that increased intracellular pH is not a necessary condition for P-gp drug extrusion activity. However, we consider that this research has a conclusion bias because it only proves acid extrusion but not drug extrusion. Actually, this research showed that P-gp seems to have proton extruding abilities.

Coley *et al.*^[112]^ found that drug resistance was related to high electric conductivity. Conductivity is not directly related to pH because it depends on the total ions (including hydrogen ions) in a solution, while pH depends only on hydrogen ions.

Mulhall *et al.*^[113]^ showed a strong inverse relationship between conductivity and initiation of apoptosis. Thus, increased cytoplasmic conductivity seems to increase the apoptosis resistance found in resistant cells.

From these two last publications, we can deduce that the “ideal” drug-resistant cells seem to be those with:

(1) high electrical conductivity; and

(2) markedly elevated cytoplasmic pH.

These “ideal” resistant cells are refractory to apoptosis induction. We can also speculate, at this point, that increased intracellular pH is not mandatory for P-gp drug extruding activity, but it is a valuable resource for resistance to apoptosis.

Confirming the importance of conductivity, it was found that the cystic fibrosis transmembrane conductance regulator (CFTR), another member of the ABC family, decreases plasma membrane electrical potential when it is over-expressed and at the same time generates an MDR phenotype, but with a low intracellular pH. There are some similarities between this regulator and P-gp and a striking difference regarding pH^[114]^.

NHE1 activity has been found to be increased in many tumors, and its downregulation re-sensitized cells to chemotherapy drugs^[115,116]^. Drug-induced cellular surface tension modifications can impact P-gp activity^[117]^.

Surface tension (interfacial tension) causes membrane rigidity^[118]^; thus, pH plays a fundamental role in this phenomenon. Furthermore, high pHi increases membrane lipid electric charges. Phosphatidylethanolamine, a normal component of the cell membrane, is involved in acid-base equilibrium with the medium.

There is abundant evidence showing that modifying cell membrane fluidity (rigidity) can reduce P-gp activity, thus reversing MDR^[119-124]^. Surfactants that reduce surface tension, such as Tween 80 (polysorbate 80), are able to reduce P-gp activity^[125]^ and improve drug delivery into the cell ^[126]^.

## HYPOTHESIS/THERAPEUTIC PROPOSAL

Based on the evidence discussed above, a triple approach against MDR is proposed here. This consists of a known P-gp inhibitor such as verapamil associated with a surfactant and a pH gradient reversal scheme.

### Verapamil

Verapamil, a calcium channel blocker**, **was first found to be an inhibitor of MDR in 1981^[127]^. It is now a well-known P-gp inhibitor that impedes P-gp protein expression at the transcriptional level ^[128]^ and increases ATP consumption in MDR cells^[129]^. Direct binding of verapamil to P-gp has also been described^[130]^. There is abundant evidence about this drug’s impact against MDR^[131-137]^.

### The surfactant Tween 80

As mentioned above, surfactants reduce cell membrane rigidity, thus counteracting one of the tools employed by MDR proteins to reject chemotherapeutic drugs. In this regard, surfactants reduce chemoresistance^[138]^. There is also abundant evidence concerning Tween 80’s anti-MDR properties^[139-142]^.

### The pH gradient reversal scheme

This scheme is based on five drugs that target different cell membrane proteins involved in pH homeostasis and in the inverted pH gradient. Its objective is to partially downregulate all the participants in the pH gradient inversion. Full blown inhibition of all of them would be impossible without serious undesired consequences for normal cells. However, partial inhibition is possible with no toxicity. These pH modulators are:

(a) amiloride;

(b) acetazolamide;

(c) lansoprazole;

(d) quercetin; and

(e) topiramate.

The appropriate combination of these drugs creates an important decrease of the intracellular pH and at the same time increases extracellular pH. Targeting pH alterations in cancer is becoming a valid strategy in complementary treatments^[143]^.

**Figure 5:**
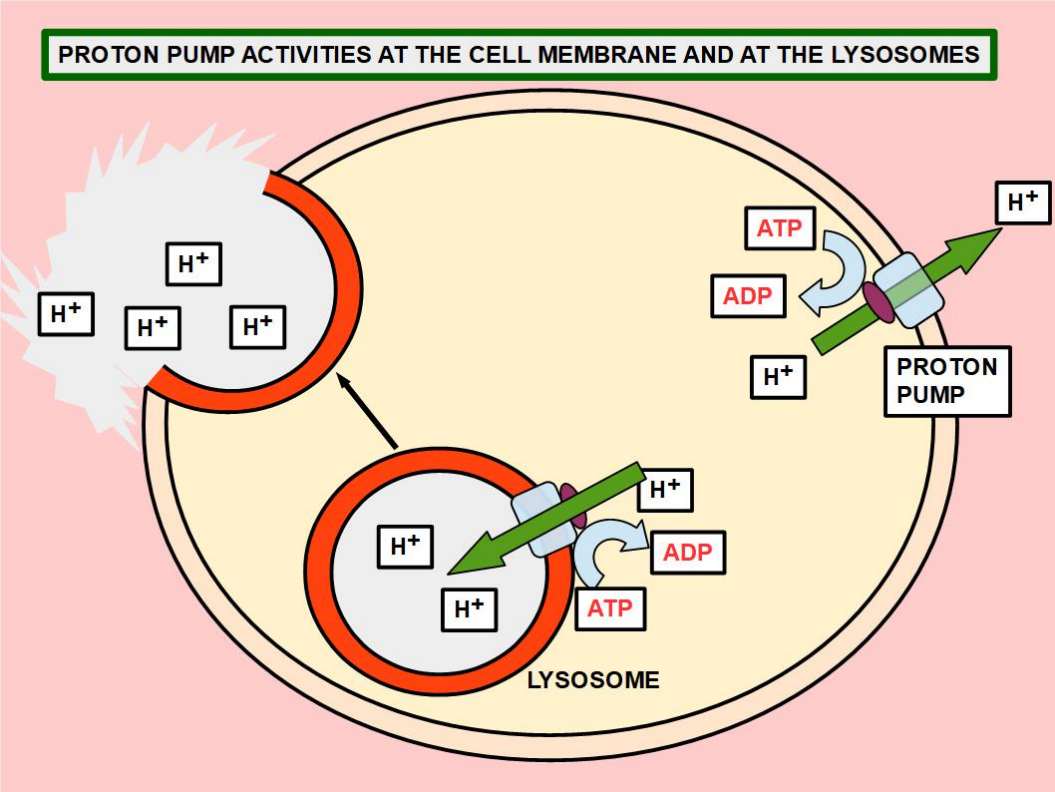
Different arrangements of proton pumps in the cell membrane and lysosomes. While in the cell, membrane the pump extrudes protons towards the extracellular space, in the lysosome, it pumps the protons into it. In a further step, the lysosome releases protons into the matrix. The functional end result is the same in both cases: inversion of the pH gradient.

• Amiloride is an FDA-approved potassium-saving diuretic in clinical use for the treatment of cardiovascular diseases and is usually associated with other diuretics such as hydrochlorotiazide. Amiloride’s main objective in the scheme is the inhibition of NHE1. Although it is a weak NHE1 blocker, at clinical doses, it is the only available approved drug. There are more potent NHE1 inhibitors; however, they are neither on the market nor FDA-approved. Evidence supporting amiloride’s anticancer effects is abundant^[144-150]^ and involves actions derived from its intracellular acidifying properties as well as its ability to inhibit urokinase-type plasminogen activator (uPA)^[151-154]^. In addition, amiloride decreases the release of tumor exosomes^[155-158]^. This exosome inhibition also reduces proton discharge and blocks an important pathway of cancer cell communication. Specifically, amiloride and its derivatives reversed MDR in different types of tumors^[159-162]^.

• Acetazolamide is a nonspecific carbonic anhydrase inhibitor. Cytoplasmic pH lowering is a known effect of this diuretic^[163-165]^ that has been in medical practice for over sixty years and is FDA approved for uses not related to cancer. There is also evidence of its ability to slow cancer growth^[166,167]^ and inhibit MDR^[168]^. Furthermore, Zheng *et al.*^[169]^ found that MDR in some tongue cancers was not produced by the three known MDR proteins of the ABC family, but rather by over-expression of CAIX. When CAIX was downregulated by antisense oligonucleotides or acetazolamide, the tumor was re-sensitized. Kopecka *et al.*^[170]^ showed that the other membrane carbonic anhydrase, CAXII, physically interacted with P-gp on the cell surface. Silencing CAXII or inhibiting it with acetazolamide created a low intracellular pH that altered P-gp’s ATPase activity and promoted chemosensitization in MDR cells. There is active ongoing research for specific CAIX and CAXII inhibitors that would make it possible to circumvent the side effects of acetazolamide^[171]^. For the time being, and until these new molecules are approved, we can only count on acetazolamide as a CA inhibitor.

• Lansoprazole is a vacuolar ATPase proton pump inhibitor approved by the FDA for the treatment of diseases related to excessive gastroduodenal acid production. At the cellular level, lansoprazole has the ability to inhibit proton extrusion from the cell, thus acidifying the intracellular milieu. Proton pumps can be found in intracellular membranes and the cell membrane. Those located in lysosomes keep the intra-lysosomal space acid while removing protons from the cytoplasm [Figure 5].

Lansoprazole was able to induce apoptosis in breast cancer cells^[172]^. Regarding MDR, lansoprazole reversed it in pets^[173]^. Other proton pump inhibitors, such as omeprazole, pantoprazole, and esomeprazole, showed incremental effects on different chemotherapeutic drugs^[174-176]^. Unfortunately, there are also negative findings, e.g., pantoprazole in a clinical trial for docetaxel in metastatic castration-resistant prostate cancer showed no effects^[177]^, despite the favorable results in laboratory level cell tests^[178-179]^. Pantoprazole even increased tumor growth and decreased chemotherapeutic cytotoxicity in mice^[180]^. The benefits or disadvantages of pantoprazole remain controversial. According to Wang et al.^[181]^, proton pump inhibitors increased chemosensitivity and improved overall survival and progression-free survival in patients with advanced colorectal cancer. It is possible that proton pump inhibitors are not all the same regarding their anticancer effects. This is the reason we choose lansoprazole, which is less controversial than pantoprazole. Proton pump inhibitors decreased cisplatin sequestration in endosomes that were finally released from the cell in a melanoma model^[182]^. Luciani *et al.*^[183]^ treated malignant cells with proton pump inhibitors, improving the accumulation of intracellular cytotoxic drugs. Intermittent proton pump inhibitors associated with standard chemotherapy administration improved the clinical outcome in metastatic breast cancer patients^[184]^.

• Quercetin is a natural flavonoid that is not approved as a drug by the FDA but is available as an over-the-counter nutritional supplement. However, it is the only compound that can be found on the market with a strong ability to inhibit monocarboxylate transporters^[185,186]^. MCT inhibition by quercetin induces important intracellular acidification^[187]^. Significantly, there is considerable evidence of its capacity to reverse the MDR phenotype^[188-218]^. Despite this large body of evidence, absolute lack of toxicity, and low cost, we could not find clinical trials exploring the substance’s ability for MDR reversal. Adverse events using high levels of quercetin (1 g daily) as a nutritional supplement have been rarely reported^[219]^. Quercetin also has additional beneficial effects in cancer:

(a) Inhibition of the PI3K/Akt/mTOR pathway^[220,221]^;

(b) Proteasome inhibition that leads to mTOR inhibition and autophagy^[222,223]^;

(c) Decrease of ROS that diminishes PKC activity^[224]^;

(d) Cancer cell-specific inhibition of the cell cycle^[225,226]^;

(e) Downregulation of heat shock protein 90^[227]^;

(f) Inhibition of β-catenin signaling^[228]^;

(g) Inhibition of pleiotropic kinases^[229]^; and

(h) Induction of apoptosis^[230]^.

• Topiramate is an FDA-approved drug for the treatment of seizures and epilepsy. It has four pharmacological effects that can benefit MDR reversal: (1) carbonic anhydrase inhibition; (2) intracellular acidification; (3) inhibition of voltage-gated sodium channels; and (4) inhibition of aquaporin 1^[231-235]^. There are no publications on topiramate having direct effects on MDR; however, refractory epilepsy in rats has been found to be associated with increased expression of P-gp^[236,237]^. Topiramate and other anticonvulsants are substrates for P-gp. Although there is no empirical proof, we suspect that topiramate may saturate P-gp extrusion capacity. The reason for including topiramate in the scheme is mainly for two of its effects: cytoplasmic acidification and voltage-gated sodium channel inhibition.

• Statins are inhibitors of the *de novo *synthesis of cholesterol by blocking hydromethyl glutaryl coenzyme A reductase, an enzyme that is a rate-limiting factor for mevalonate synthesis and the mevalonate pathway. Therefore, statins decrease endogenous cholesterol production. Cell membrane rigidity depends on the amount of cholesterol, among other factors.

## DISCUSSION

pH homeostasis is a complex mechanism in which different transporters, exchangers, channels, and enzymes are involved in overlapping proton and ionic trafficking between different cellular compartments. The results of these ionic movements lead to the best possible pH balance for cellular functions. Tumor pH homeostasis is different from that found in normal tissues, and this difference involves a proliferative and progressive advantage for the malignant phenotype. Each enzyme in a complex organism has a specific pH in which it works at the optimum speed and capacity. This is the pK. Tumors, by creating a different pH homeostasis, are signaling which enzymes should be more active and when, thus regulating tumor metabolism.

This essentially means that pH is a signaling molecule. If anyone doubted that pH is a molecule, he or she would be right. It is not a molecule but many molecules, or, even better, many protons. The cell behavior is thus conditioned by the number of protons present. The MDR phenotype shows a slight difference with the drug-sensitive one: a higher intracellular pH. This difference allows for two characteristics of the MDR cell:

(1) A more rigid cell membrane that plays a role in impeding cytotoxic drug access inside the cell; and

(2) A higher resistance to apoptotic signals.

A third characteristic must be added to this: the ion trapping produced by the strongly acidic extracellular matrix.

Multidrug resistance is not a one-protein job. At a certain point, P-gp and its sister molecules of the ABC family require adequate cell membrane rigidity, higher apoptosis resistance, and more ion trapping, whether in the matrix or inside lysosomes. This means the appropriate pH. MDR seems to function better with a high intracellular pH. This does not mean that one is the cause of the other. An MDR phenotype can be achieved even with low intracellular pH; this is the case of CFTR. Conversely, a high pHi can generate an MDR phenotype without over-expressing the MDR proteins.

This evidence hints towards the idea that high pHi and the MDR proteins complement each other, rather than there being a causal relationship. Both come together in one characteristic of the resistant cell: increased cell membrane rigidity. High pHi induces membrane rigidity, which in turn cooperates with MDR.

All this said, it becomes evident that for a successful fight against MDR, it is not enough to downregulate P-gp, etc., but pH and cell membrane rigidity must be tackled as well. The scheme proposed here confronts the three issues:

• P-gp with calcium channel blockers such as verapamil or others;

• pH gradient inversion with the pH-centered treatment; and

• membrane rigidity with surfactants such as Tween 80 and others.

The MDR problem has even further complexities. Balza *et al.*^[238]^, working with two different breast cancer cells, a triple-negative one and a hormone-sensitive one, found that:

(1) Associating cisplatin with an amiloride derivative was significantly more effective than treatments with cisplatin plus esomeprazole in triple-negative cells; and

(2) Esomeprazole alone was more effective in hormone-sensitive cells.

This shows that pH-centered treatments as complementary therapy may differ according to the type of cell.

Importantly, persistent intracellular acidification was able to downregulate the MDR phenotype^[84]^.

**Figure 6:**
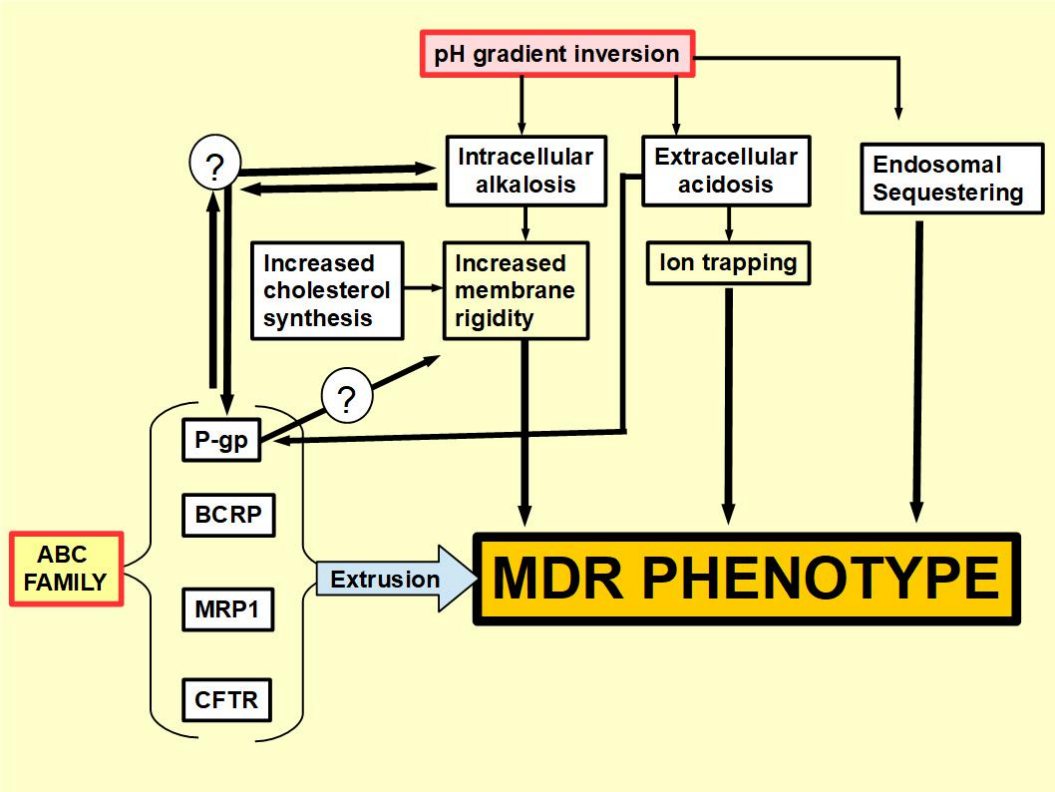
A synthesis of known and unknown relationships between pH and the MDR phenotype. Based on references cited above (Ref.^[48-50,239-241]^). Multiple drug resistance (MDR).

**Figure 7:**
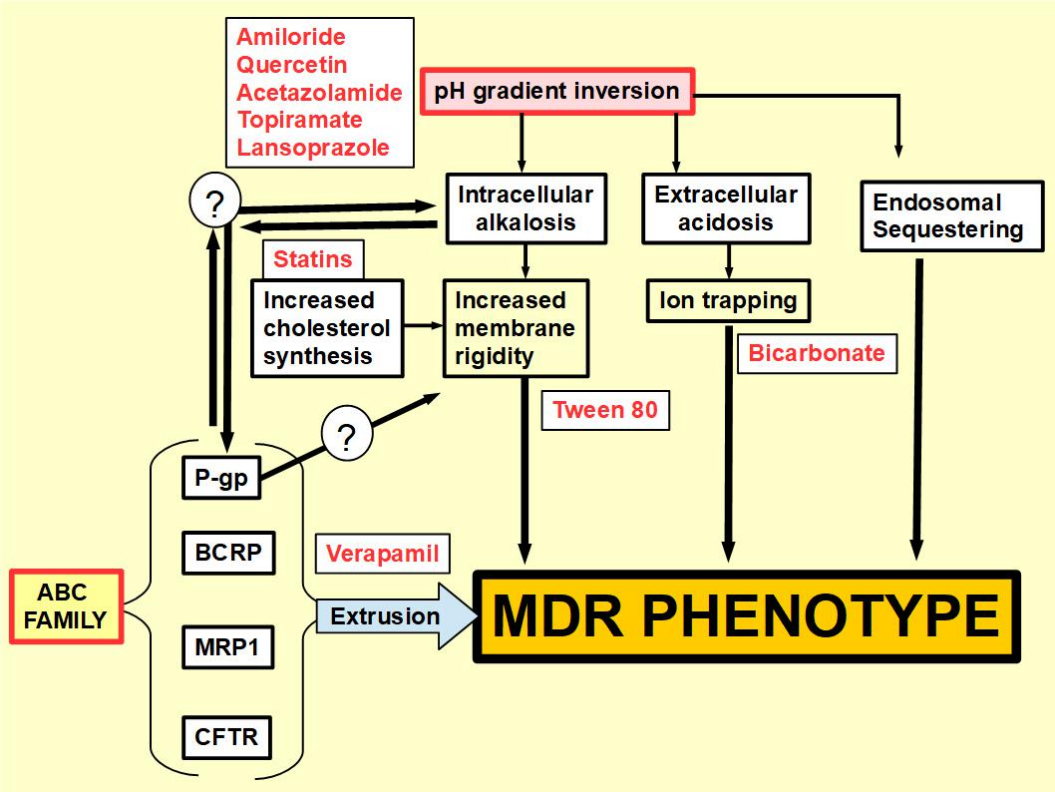
Site of actions of the anti-MDR scheme. Reversion of the deregulated pH gradient is able to act against the MDR phenotype in two ways, by decreasing extracellular acidity and reducing intracellular pH. MDR: Multiple drug resistance.

Figure 6 is a summary of concepts discussed in this paper, while Figure 7 shows the site of action of MDR inhibitors.

It is important to note that extracellular acidity per se can induce P-gp expression. Figure 6 shows that the ABC family of drug extruders, intracellular alkalosis, and extracellular acidosis can all generate an MDR phenotype in an independent manner. However, there is evidence supporting the relationships among these three factors. Increased extracellular acidity induces P-gp expression. MDR cells with increased P-gp expression usually show increased intracellular alkalinity^[87,95]^. This, in turn, prevents apoptosis and increases cell membrane rigidity^[112,113]^, creating the ideal environment for drug resistance^[117]^.

## CONCLUSIONS

MDR represents the last chapter of chemotherapeutic cancer treatment. It leaves the oncologist on a very narrow path to continue patient care. Unfortunately, there is no accepted treatment protocol. In this review, we propose a multidrug approach that simultaneously targets three important MDR characteristics, namely the MDR proteins, dysregulated pH, and cell membrane rigidity, with a rationally constructed approach.

This scheme has not been tested on clinical grounds. However, each of its components has separately provided successful experimental results, with the exception of topiramate, which has not been tested in the MDR context. This justifies their combination, as each of them targets different aspects of the MDR conundrum. Well-planned clinical trials are needed to evaluate this proposal. Furthermore, the scheme has almost no toxicity for normal cells, and there is ample clinical experience with the use of all these drugs.

## DECLARATIONS

### Copyright

© The Author(s) 2022.
